# Supplementary material for: Long-Term Therapeutic Effects of 225Ac-DOTA-E[c(RGDfK)]2 Induced by Radiosensitization via G2/M Arrest in Pancreatic Ductal Adenocarcinoma
Source: Pharmaceutics. 2024 Dec 24;17(1):9. doi: 10.3390/pharmaceutics17010009 (PMC11768328; doi:10.3390/pharmaceutics17010009)
Supplement: Supplementary file 1 [file pharmaceutics-17-00009-s001.zip › pharmaceutics-3371007-supplementary.pdf]

Supplementary Materials

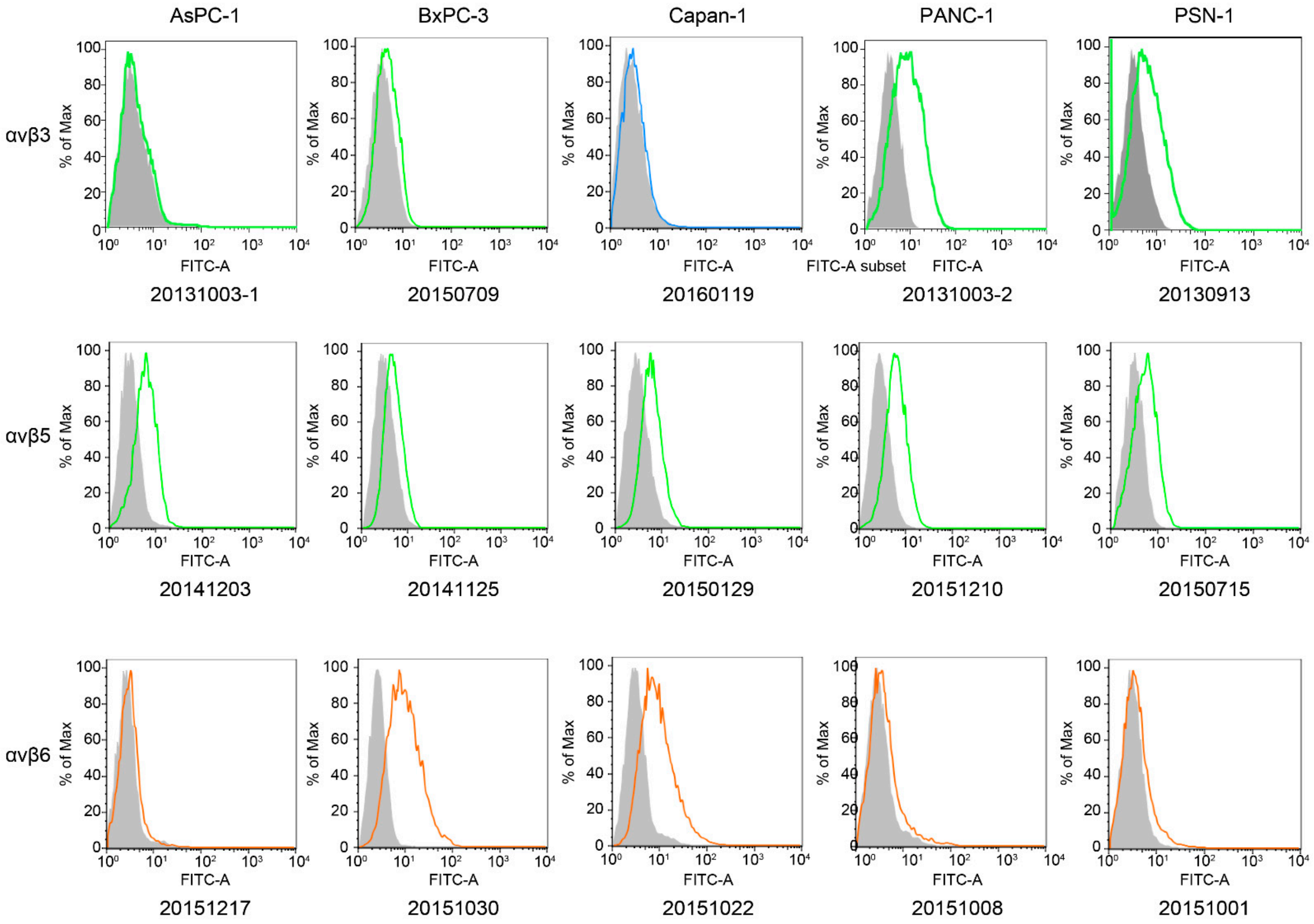

Figure S1. Expression of $\alpha_v\beta_3$, $\alpha_v\beta_5$, and $\alpha_v\beta_6$ in PDCA cell lines.

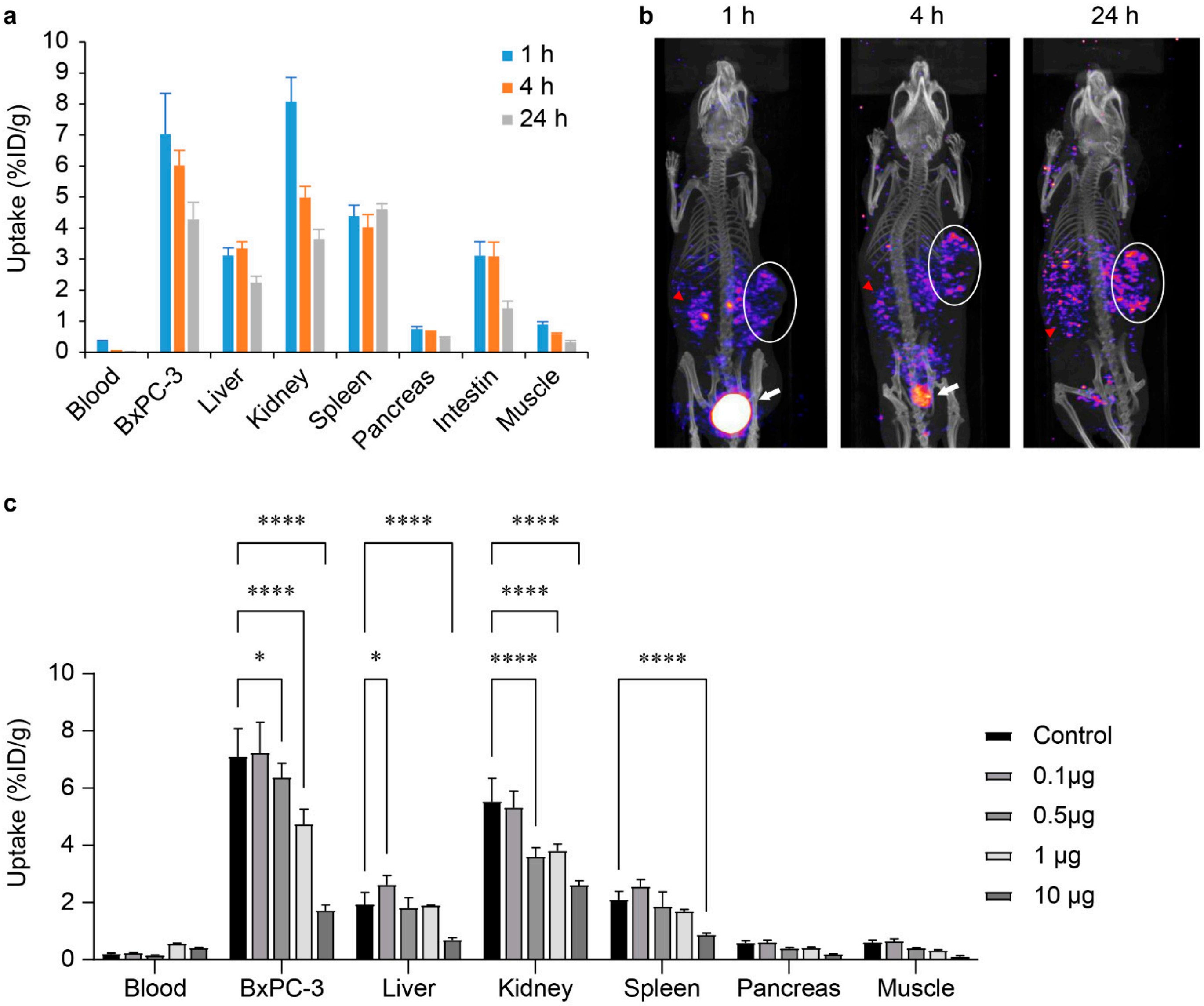

Figure S2. Pharmacokinetics of ^{111}In-DOTA-RGD$_2$ in BxPC-3 xenograft model.

a Biodistribution of ^{111}In-DOTA-RGD$_2$; **b** SPECT/CT images. The white circles indicate tumors, the red arrowheads indicate kidneys, and the white arrows indicate bladders. **c** Biodistribution with co-injection of DOTA-E[c(RGDfK)]$_2$.

(* $p < 0.05$, **** $p < 0.0001$)

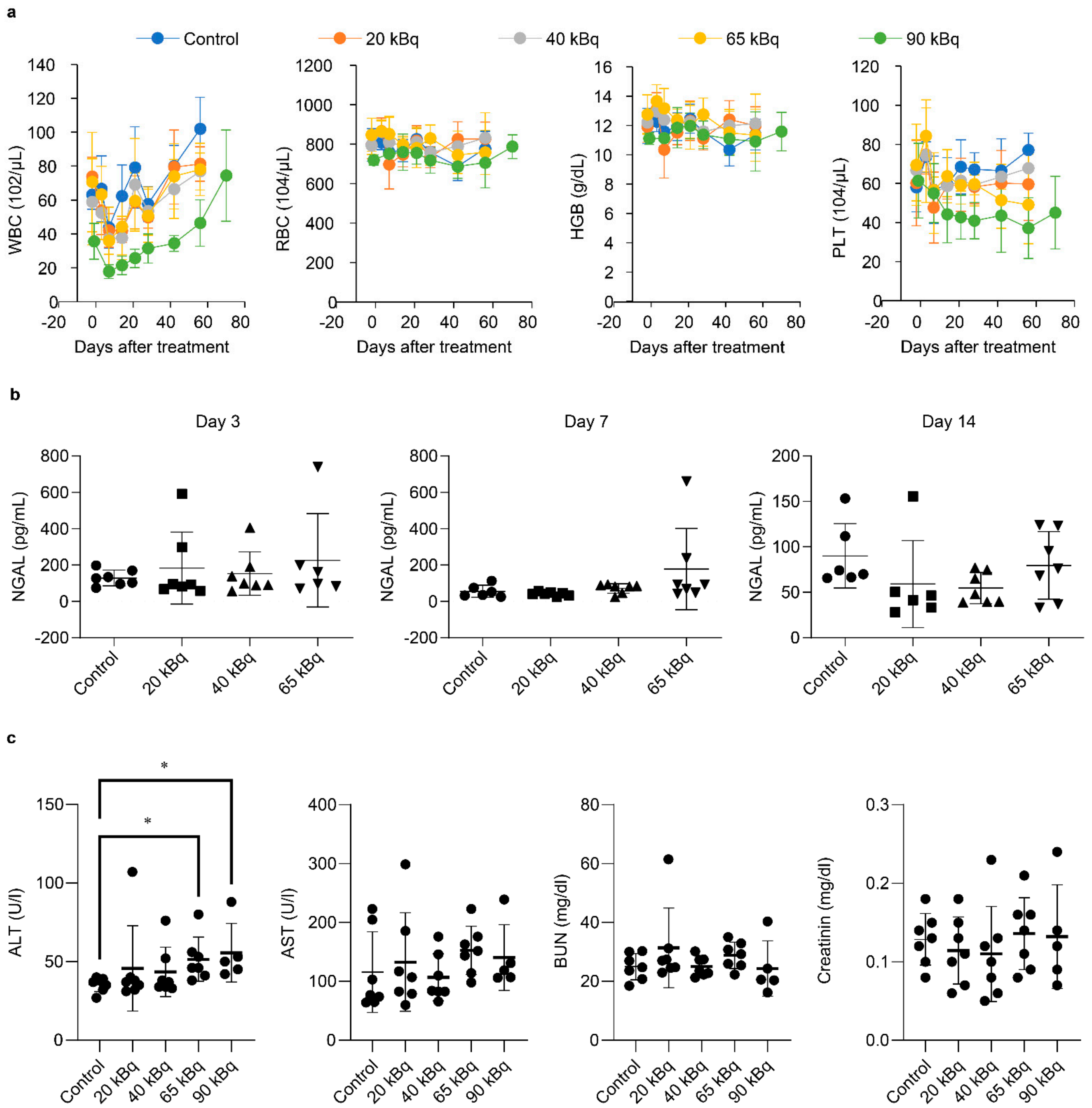

Figure S3. Toxicity evaluation after treatment with ^{225}Ac-DOTA-RGD$_2$.

a. The peripheral blood counts were monitored at the indicated date. Data represent mean ± SD. WBC, white blood cells; RBC, red blood cells; HBG, hemoglobin.; PLT, platelets. **b.** NGAL was evaluated to investigate acute kidney injury by ^{225}Ac-DOTA-RGD$_2$ (n = 6–7). **c.** ALT, AST, BUN, and creatinine were measured at the end of the study.

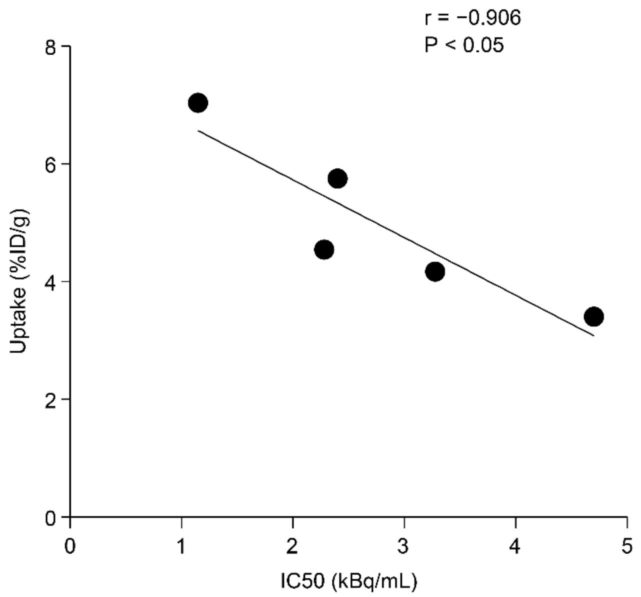

Figure S4. Correlations between the in vitro cytotoxicity (IC_{50}) and tumor uptake of ^{225}Ac-DOTA-RGD_2 (%ID/g). Pearson's correlation coefficient was calculated using GraphPad Prism 9 (r = −0.906, $p < 0.05$).
